# Ambulatory pediatric cochlear implantation: safety, efficacy, and feasibility – first multicentric Moroccan study on 681 children

**DOI:** 10.1186/s12887-025-06169-4

**Published:** 2025-10-06

**Authors:** Amal Hajjij, Khadija Elbouhmadi, Oussama El Gamali, Anas Benjilali, Sara Benchikh, Amal Haoudar, Said Anajar, Loubna Taali, Chafik Elkettani, Khalid Snoussi

**Affiliations:** 1 Mohammed VI University of Sciences and Health (UM6SS), Casablanca, Morocco; 2 Mohammed VI Audition Center, Department of Otolaryngology Head and Neck Surgery, Mohammed VI Foundation of Sciences and Health (FM6SS), Casablanca, Morocco; 3 Mohammed VI International University Hospital & Cheikh Khalifa International University Hospital, Casablanca, Morocco; 4 Department of Anesthesiology, Mohammed VI Foundation of Sciences and Health (FM6SS), Casablanca, Morocco; 5 Laboratory of Physiopathology and Molecular Genetics, Faculty of Sciences Ben M’Sik, Hassan II University, Casablanca, Morocco

**Keywords:** Day surgery, Otology, Cochlear implantation, Pediatrics

## Abstract

**Objective:**

Our aim is to evaluate the safety, efficacy and feasibility of outpatient cochlear implantation in children.

**Study design:**

Multicentric retrospective study analyzing 681 children who underwent cochlear implantation from October 2016 to May 2024.

**Setting:**

Ear, Nose, and Throat departments of Mohamed VI Foundation of Sciences and Health, Morocco.

**Methods:**

In this report, we examined the protocol used, anesthetic and surgical techniques, postoperative data and success rate of ambulatory cochlear implant surgery. Data collection included demographic information, surgical and postoperative results.

**Results:**

A total of 721 cochlear implants were performed between October 2016 and May 2024, including 681 in children and 40 in adults. Of these children, 674 were included, of whom 661 (98%) benefited from outpatient surgery and 13 (2%) required overnight hospitalization. Conversion to overnight hospitalization was required for 8 patients who suffered post-operative vomiting, requiring medical management and intravenous infusion, and for 5 patients who suffered post-operative dizziness, requiring in-hospital monitoring.

The median age of our patients was 3.7 ± 2.4, with a predominance of males (sex ratio = 1.46) and patients coming from all regions of Morocco.

Severe to profound bilateral hearing loss was noted, and in the majority of cases, imaging was normal. Surgeries were performed without major complications, but short-term complications did occur, such as vomiting in 8 patients and vertigo in 5. The satisfaction rate was 100%, the success rate 98% and the failure rate 2%, with no readmissions.

**Conclusion:**

Our results demonstrate the efficacy and safety of outpatient cochlear implant protocol for pediatric population in our setting.

## Background

Ambulatory or “day” surgery is a planned surgical procedure carried out in a fully equipped operating room, enabling the patient to return home the same day without having to prolong his hospital stay [15]. This approach is attracting growing interest in many medical fields. According to the United States National Health Statistics Reports, an estimated 48.3 million ambulatory procedures, both surgical and nonsurgical were conducted in hospitals and ambulatory surgery centers (ASCs) in 2010 [12].

The benefits of this procedure include lower hospital costs, reduced risk of nosocomial infections, significantly reduced waiting times, low complication rates and it allows patients to recover in the comfort of their homes [5].

In Morocco, literature reports on ambulatory surgeries are quasi-absent, with only one study conducted at the Surgery Department of Hassan II University Hospital on patients undergoing procedures for hernial, gallbladder stone, and various proctologic pathologies over a six-year period [24].

In Otologic surgery, outpatient procedures vary from one center to another [19]. A US study of 16,709 outpatient otologic surgeries revealed that the most common procedures in their adult patient cohort were tympanoplasty (47.4%), stapedectomy (15.0%), cochlear implantation (8.6%), tympanomastoidectomy (4.3%) followed by mastoidectomy (4.2%) [13].

Outpatient cochlear implant surgery in pediatrics is considered an effective approach to treating severe to profound deafness in young children [5].

The purpose of our study is to report the experience of ENT departments of Mohamed VI Foundation of Sciences and Health in outpatient pediatric cochlear implantation surgery. We aim to assess the feasibility and safety of this approach, as well as to analyze the results obtained in our pediatric patients.

This study is the first one in Morocco to report results on outpatient cochlear implant surgery in children.

## Patients and methods

We conducted a multicenter retrospective study including 681 of children operated for cochlear implants between 2016 and 2024 at the ENT departments of Mohamed VI Foundation of Sciences and Health in Cheikh Khalifa International University Hospital and Mohamed VI International University Hospital. Afterwards, all patients were followed in the Mohamed VI Hearing Center affiliated to the same foundation.

### Inclusion criteria

We included in our study patients from 9 months to 16 years with severe to profound congenital or acquired bilateral deafness with an indication for cochlear implantation, after adequate audiological testing, psychological evaluation, CT and MRI imaging. We also considered patients with no specific physical conditions that could complicate anesthesia, nor foreseeable anatomical variations that could cause complications; we included patients with a complete clinical file including and a detailed operation and hospitalization report.

### Exclusion criteria

Infants under 9 months of age were excluded because of the increased risk of sudden death, ex-premature during their first year of age and cardiac malformations requiring prophylactic antibiotic therapy. In addition, patients with incomplete medical records, an inability to undergo outpatient treatment, who lived more than 25 km from the hospital, whose parents were illiterate or had no means of communication with the hospital, and who had a history of postoperative nausea and vomiting (PONV) following previous surgery were omitted from the study.

### Data collection and analysis

Clinical data were collected first on paper and then electronically via the institutional application DxCare SIH-HCK version 7.7-7p059 (Dedalus, France). Epidemiological, clinical, paraclinical and intraoperative data were collected for all patients.

Data was entered into Microsoft Excel and analyzed with RStudio version 4.3.1. In addition, qualitative and quantitative parameters, outpatient success rate, failure rate and readmission rate were calculated. Satisfaction questionnaires were also used: an ambulatory questionnaire detailing preoperative guidelines, a postoperative questionnaire administered by telephone to assess aftercare and pain management, and an ambulatory surgery passport to facilitate understanding of the patient circuit and ensure that the necessary documents were complete.

### Definition of parameters studied: Success rate, Failure rate, complications occurring immediately (short-term) after the procedure

The key parameters of ambulatory surgery were defined as follow: the success rate corresponds to the percentage of patients leaving the hospital the same day after surgery. The failure rate is the percentage of patients with post-operative medical complications who require hospitalization. The readmission rate is the rate of patients with complications (hemorrhage, infection, pain, etc.) in the days following the operation and require readmission to hospital after being discharged. It should be noted that we considered short-term complications that happen immediately or the days following the operation. These can include bleeding, hematoma and infection at the incision site, nausea and vomiting, dizziness and fainting.

### Medical protocol

#### Anesthesia

##### Pre-anesthetic evaluation

Prior to the operation, a pre-operative assessment by an anesthesiologist included a complete review of the birth, medical, surgical and family histories; a review of the medical record; evaluation and review of laboratory, radiologic, and other investigations; and physical examination.

##### Pre-operative Preparation

On the day of surgery, the patient had to fast for 6 h for solids and infant formula, 4 h for breast milk and, 2 h for clear liquids. Also, patients coming from other cities had been admitted a day before.

##### Admission to the operating room and monitoring

All patients were identified, and consent was verified before their entry to the operation room. Monitoring was done with electrocardiography, pulse oximetry, EtCo2, and noninvasive blood pressure monitoring. All basal parameters were recorded. Warming blankets were used to avoid hypothermia.

##### Anesthesia in the operating room

The inhalation induction by sevoflurane was the most common technique used in our setting. IV induction was reserved for older children and those with a previously established IV catheter, in this case, anesthesia induction was done with propofol 2,5–3,5 mg.kg^−1^ intravenous mixed with lidocaine (0.5 to1 mg/kg) to attenuate the pain related to propofol administration. The maintenance of anesthesia was done with end-tidal 1.5 MAC sevoflurane in 2:1 ratio mixture of air and oxygen; neuromuscular blockade was reserved for older children. For analgesia, a single dose of fentanyl 2 mcg/kg was given. Intubation was done with an appropriate-sized endotracheal tube and checked for bilateral air entry and the tube was secured firmly in place. Patients were placed in mastoidectomy position. For PONV, we had given ondansetron 0.1 mg/kg and dexamethasone 0.15 mg/kg combination. General anesthesia lasted for an average of one hour and half.

### Surgery

The cochlear implantation procedure was minimally invasive. Primarily it began with retro auricular incision followed by an elevation of triangular musclo-periostal flap and minimal musculoperiostal pocket with minimal tissue dissection. It allows to access the mastoid bone which will be followed by a restrictive mastoidectomy to access the middle ear through a drilled opening in its posterior bone wall called posterior tympanotomy. The round window approach was chosen for all patients when possible. Once the round window membrane was accessible, a small opening was made to insert the electrode of the cochlear device. The internal device containing the stimulator receiver was placed on a drilled bed under the musculo-periostal pocket before the insertion of the electrode through the round window approach into the cochlea. Finally, the incision behind the ear was closed after a careful suture of the musculo-periostal layer and a gently tight bandage was applied. Intraoperative objective measurements, i.e. impedance, and NRTs, were measured for all our patients.

### Hospital management of ambulatory care

To ensure the safety and well-being of patients, postoperative monitoring in a post-interventional monitoring room (PIMR) was an essential part of ambulatory care management. Before leaving the hospital, the patient must meet precise criteria, known as discharge criteria. Firstly, physiological parameters were assessed and must be normal. The patient able to walk should stand unaided, to feed by mouth, and there must be an absence of nausea, vomiting, dizziness and pain. Patients and their relatives received post-operative instructions and prescriptions for post-operative care before discharge. Anesthetists were available to patients in case of immediate need by telephone after discharge. The hospital telephone number is also provided in case of emergency. The patient must be within a 25 km radius of the hospital and will receive a call in the morning to assess his condition.

### Ethical considerations

In this study, each participant explicitly consented to the anonymous use of their data for research purposes, adhering to the ethical principles outlined in the Declaration of Helsinki. This retrospective study did not modify patient management and did not require prior approval from the ethics committee.

## Results

In this section, the demographics of our patients were distributed by age, gender and geographic origin, followed by a brief overview of the results of the pre-implant evaluation, including our patients’ audiological data, radiological findings and speech therapy assessments, surgical procedure and post-operative follow-up. Performance indicators such as success, failure and readmission rates were highlighted. Finally, the results of the post-operative satisfaction questionnaire were analyzed.

From October 2016 to May 2024, we performed a total of 721 implants, including 681 in children and 40 in adults (aged over 16).

Of the children, 8 had to spend the night in the hospital and were therefore not treated as outpatients. These scheduled hospitalizations were due to specific reasons: 4 cases for social reasons or distant geographical location, and 4 cases for malformations previously diagnosed by CT scan.

**Fig. 1:**
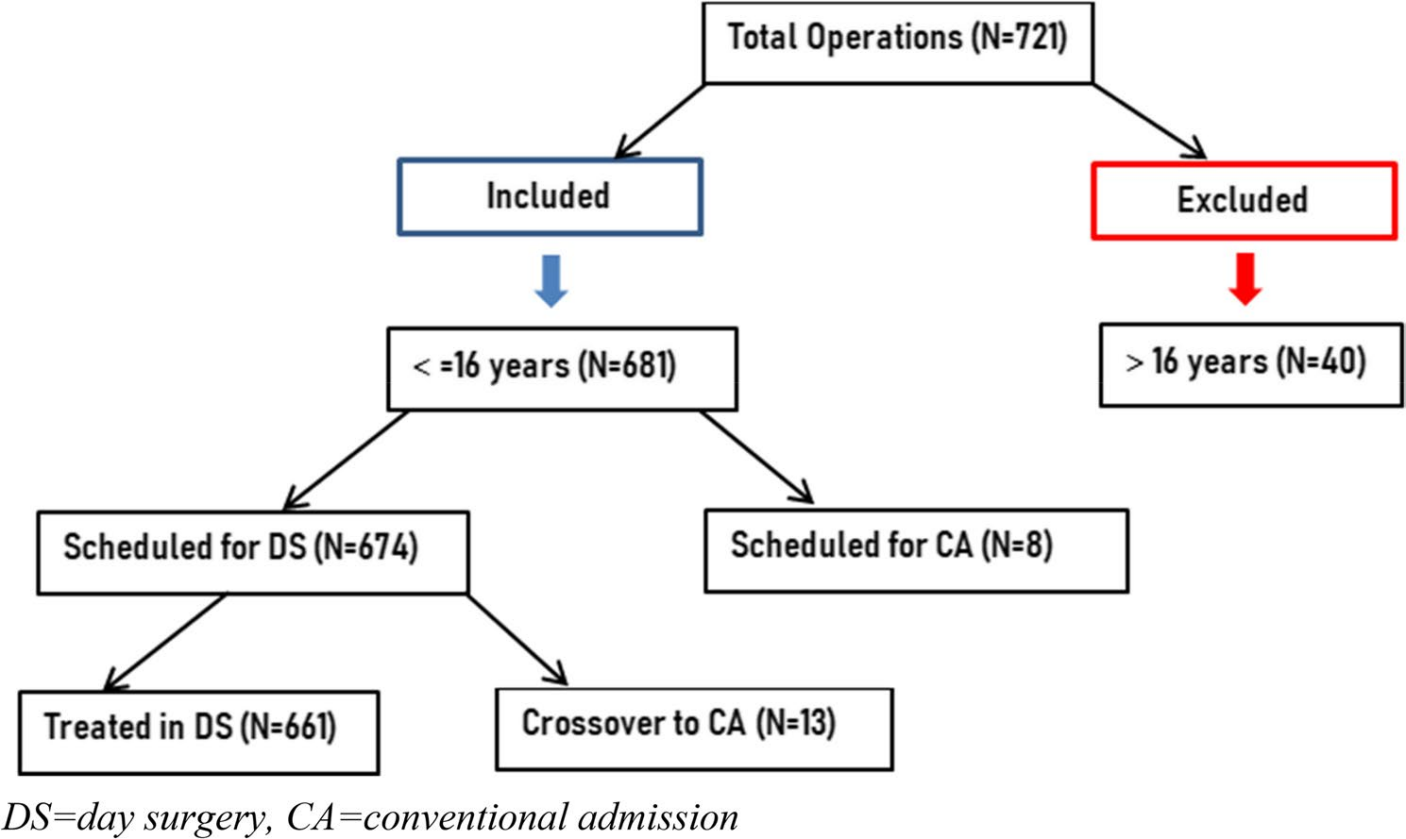
Distribution of cochlear implant patients received in our Center

In all, 674 children were included in the study and have received outpatient treatment. Of these children, 661 (98%) were actually managed as outpatients. The remaining 13 (2%), initially scheduled for outpatient treatment, ended up spending the night in the hospital (Fig. 1). Patients who were initially scheduled for day surgery but subsequently required admission were usually discharged the day after surgery.

The mean age of our pediatric population is approximately 3 years and 8 months, with a standard deviation of approximately 2 years and 4 months (Table 1). The ratio of 1.46 suggested a male preponderance in our overall cohort. The geographical origin of patients showed a diversified distribution across Morocco, with strong representation from areas such as Casablanca, Fès-Meknès and Tangier. A significant proportion of patients also came from other urban or rural regions of Morocco, at a rate of 1 or 2 per region (Table 1).

**Table 1:** Demographic profile of our pediatric patients

Demographics	
Mean Age ± SD	3.7 ± 2.4
Sex-Ratio	1.46
Geographic origin	Patients (N)
Casablanca	103
Fès-Meknès	78
Tangier	58
Rabat-Sale	27
Marrakech	20
Kenitra	17
Nador	17
Tiznit	16
Alhoceima	15
Eljadida	14
Taouant	12
Tetouan	11
Taza	8
Oujda	8
Temara	7
Ouazane	7
Berrechid	5
Zagoura	4
Midelt	4
Beni Mellal	4
Berkane	4
Mohamadia	3
Unknow	94
Other parts of Morocco	145

In terms of etiology, in patients implanted after the age of 5 years, 57.6% suffered from congenital progressive hearing loss and 33% from acquired hearing loss. The most common acquired etiologies were meningitis (1.9%) fever (1.9%), prematurity (0.6%) and neonatal hypoxemia (0.1%) (Table 2).

**Table 2:** Acquired hearing loss etiologies in post-5-year cochlear implant patients

**Etiology**	**Number of cases (% of total)**
Meningitis	20 (1.9%)
Fever	20 (1.9%)
Prematurity	6 (0.6%)
Neonatal hypoxemia	1 (0.1%)

The indication for cochlear implantation was based on the results of auditory brainstem responses (ABR) and behavioral audiological assessment showing severe to profound bilateral hearing loss. Neuroradiology evaluation, including CT and MRI scans of the temporal bones, revealed a normal appearance with presence of the cochleovestibular nerve, absence of major cochlear malformations in the majority of patients.

All patients received the same anesthetic method with no major complications. Minor complications included mild vertigo, one case of post intubation laryngitis and moderate vomiting.

Surgery was performed under general anesthesia without curarization, with facial nerve monitoring and without incident. A round window approach was preferred and drilling of the crista ante fenestram was only necessary if the round window membrane was not completely apparent (82%). A cochleostomy was necessary only in 2 cases.

Results showed that the majority of patients had normal impedance measurements, and almost all NRTs were normal. Complete insertion of the all electrods was achieved in 92% (620 patients). The average NRT measurement was 190.29 µV, ranging from 90 µV to 250 µV.

The average hospital stay was 8 h, with same-day discharge for patients with no complications. Patients woke up in the post-implant room, where a nurse specialized in cochlear implants checked for complications.

Conversion to overnight hospitalization was required for 13 patients: 8 due to post-operative vomiting requiring medical management and IV infusion, and 5 due to post-operative dizziness requiring inpatient hospital monitoring.

A retroauricular bandage was applied at the end of the operation. After surgery, patients left hospital the same day and stayed in Casablanca for a week. For the few patients who required hospitalisation, the length of stay was generally limited to one night, with a return home the following day. All patients returned after 7 days for a follow-up visit, during which the dressing was removed. They were then able to return to their home town and were invited back approximately one month later for implant activation.

The satisfaction rate of the patients surveyed showed that neither they nor their families were dissatisfied with their experience of outpatient cochlear implant surgery. With regard to the post-operative period, only six patients reported nausea and vomiting, four mentioned poor sleep quality, and two suffered post-operative pain which was treated medically at home. (Table 3).

**Table 3:** Post-Operative outcomes and patient satisfaction

Results	*N* (%)
Post-operative complications *Poor quality of sleep* *Post-operative pain* *Nausea and vomiting*	12 (1.8%) 4 (0.6%) 2 (0.3%) 6 (0.9%)
Satisfaction rate	674 (100%)
Success rate	661 (98%)
Failure rate	13 (2%)
Readmission rate	0 (0%)

Overall, outpatient discharges were successful, with a 98% success rate, while the failure rate was 2%. Notably, no patients required readmission, testifying to the effectiveness and safety of the discharge protocol.

## Discussion

Ambulatory surgery is a pre-programmed medical practice that enables patients to undergo surgical procedures and return home the same day (2). This approach is used for surgical procedures that do not require prolonged post-operative monitoring. It was first implemented in 1909, when the Royal Glasgow Hospital for Children demonstrated the effectiveness of ambulatory surgical care for certain procedures, marking the beginning of this innovative practice [9]. According to the American Society of Anesthesiologist (FASA), in 1980 the most frequent outpatient surgical procedures were dilatation and curettage (17.2%), myringotomy (13.4%), tubal ligation (9. 7%), orthopedic procedures (7.7%), dental procedures (5.3%), excision of skin lesions (5.0%), diagnostic laparoscopy (3.7%), tonsillectomy and adenoidectomy (3.6%), cystoscopy (3.1%) and arthroscopy (2.7%) [4].

In the United States, outpatient surgery is growing steadily, accounting for a significant proportion of surgical interventions, with a forecast of over 144 million outpatient procedures by 2023, according to recent reports [27]. The rate of ambulatory surgery in France in 2020 was 58.61% according to the French Ambulatory Surgery Association (AFCA), and by 2022 it has accelerated to over 63% nationally according to the national health agency (ARS) Nouvelle-Aquitaine.

Readmission rates reported by the Royal College of Surgeons of England, after day-surgery was 2–3% which is very low, and therefore day-surgery may be considered safe [2].

Among the benefits of outpatient surgery is enabling patients to recover within the comfort of their homes, reducing the risk of infections and reducing patient stress and anxiety. Patients can also benefit from a quicker return home to a normal life [22]. Economically speaking, procedures are faster in ambulatory surgery centers, which reduces costs and frees up more beds for other patients, thus meeting growing demand for these procedures. The savings from outpatient procedures can be reinvested in other areas of healthcare [17, 26].

In otology, minor surgical procedures include myringotomy, insertion of tympanostomy tubes and excision of small auricular lesions and are all usually performed as ambulatory procedures. Major procedures include tympanoplasty, mastoidectomy, stapedectomy, cochlear implantation, vestibular schwannoma resection, labyrinthectomy, facial nerve decompression or repair, middle ear tumor resection, mastoidectomy of the canal wall and ossicular chain reconstruction [21].

Cochlear implantation is a major inner-ear surgery performed via the middle ear where the surgeon creates a mastoidectomy then a tympanotomy to access the middle ear, then inserts the implant through the round window into the cochlea, located in the inner ear [7]. This surgery is well accepted as the treatment of choice for bilateral severe to profound sensorineural hearing loss in children [11].

It can be performed on an outpatient basis under certain conditions, such as careful patient selection, absence of foreseeable medical or anatomical malformations predicting complications, good family support, proximity to a medical center for emergencies and the patient’s ability to recover quickly from general anesthesia [20].

Our study aimed to determine the efficacy and safety of cochlear implants in an outpatient setting among 681 Moroccan pediatric patients aged 9 months to 16 years. The mean age of our patients was 3 years and 8 months, with a higher prevalence of procedures in males than in females. This correlates with the literature, which reported that deafness is more prevalent in males than in females [28]. We also noted that these patients come from various regions of Morocco, reflecting a varied representation of our pediatric population.

The selection of our patients was carried out in a rigorous manner to ensure the success of our outpatient experience and aimed to establish precise criteria including thorough evaluations.

In the existing literature, details of inclusion and exclusion criteria for cochlear implantation in children aged 17 or under in an outpatient setting are often limited.

According to Sivam et al., inclusion criteria for cochlear implants in children aged 17 or under reported in a children’s hospital and in an outpatient surgical center were patients with pre-lingual and post-lingual deafness, which are classical cochlear implant indications [23]. Another study reported the inclusion of patients admitted for the first time to the pediatric outpatient surgical unit for unilateral cochlear implantation, and required medical and surgical criteria to be met in accordance with recommendations established by the Conseil National de Chirurgie Infantile (CNCE) and the Association des Anesthésistes Pédiatriques Francophones (ADARPEF). The same study excluded children who would have to undergo surgery under conventional admission, the presence of comorbidities incompatible with day surgery and residing at a distance from the hospital center [16]. Stephens et al. have reported the inclusion of all 21 pediatric patients who underwent cochlear implantation at the Royal National Throat Nose and Ear Hospital with no mention of exclusion criteria [25].

The surgical technique used in our study involved a mastoidectomy with posterior tympanotomy to insert the electrode into the cochlea which is comparable to those on the literature. The approach described by Aldhafeeri et al. involves the use of the facial recess approach with mastoidectomy [1]. For Stephens et al., a cortical mastoidectomy with posterior tympanotomy and cochleostomy was used for cochlear implantation [25].

To compare our results with three other similar studies, we took into account the number of patients, study period, mean patient age, sex ratio and success, failure and readmission rates (Table 4).

**Table 4:** Comparison of our results with other studies on pediatric cochlear implantation

Study	Number of patients	Study Period	Mean age (years)	Sex-Ratio	Success rate (*N*,%)	Failure rate (*N*,%)	Readmission rate (*N*,%)
Our study	674	2016–2024	3.69	1.46	661(98%)	13(2%)	0 (0%)
Micalleti et al. [20]	66	2017–2022	4	0.61	57 (86%)	9 (14%)	0 (0%)
Hugel et al. [23]	190	2016–2020	-	-	181 (95.3%)	9 (4.7%)	5 (2.6%)
Boullaud et al. [24]	47	2017–2019	-	-	40 (85%)	7(15%)	0 (0%)

*N Number of patients, % percentage*

In our study, 674 children underwent an outpatient cochlear implant procedure, with a 98% success rate, a 2% failure rate and no cases of readmission. On the other hand, Micatelli’s study, carried out over a 5-year period, reported a median age of 4 years, with a female preponderance, their success rate was 86%, the failure rate 14% and no cases of readmission were reported [16]. Hugel et al. included 190 patients over a 4-year period, with a failure rate of 4.7% and a readmission rate of 2.6% [14]. According to Boullaud et al., 47 cochlear implant procedures were performed over a period of 2 years, with a reported success rate of 85%, a failure rate 15% and no readmissions [3].

Several factors explaining the predominance of male deafness have been mentioned in the literature, such as exposure to noise, as in professional contexts (industrial, military), increased vulnerability of the male sex in the perinatal period to hypoxia or prematurity, and the protective effect of oestrogen against deafness in women, which does not apply to men [8, 10, 18]. Furthermore, this predominance is not always systematic in all studies, since a higher rate of women can be found in literature.

Differences observed in the results between our study and the other studies may be explained by differences in patient demographics, sampling bias, and variations in postoperative protocols.

Moreover, we noted a positive correlation between Mondini malformations and postoperative vertigo, which translates into a higher failure rate in patients with these malformations. Given the risk of postoperative complications, it might have been preferable to exclude them from the beginning.

When it comes to outpatient care, a well-structured protocol is crucial for achieving a high success rate. In our experience, this begins with pre-admission instructions prior to the operation day, where the patient meets with the surgeon to schedule the operation date and signs a consent form. Following this, there is a consultation with the anesthesiologist and preparation of a pre-admission file, during which additional prescribed tests (X-ray, blood test, etc.) are conducted. The day before arrival, the patient receives a phone call to confirm their arrival time at the hospital. Patients are required to follow specific pre-arrival instructions, such as fasting for 6 to 8 h, showering with betadine, washing hair, and bringing all administrative and medical documents. On the day of the operation, patients proceed to the outpatient department, where they are prepared with the assistance of a nurse. After the procedure, patients are taken to the recovery room, where a stretcher-bearer transports them back to their room for monitoring. They are accompanied by the paramedical team, and the surgeon and anesthesiologist visits to provide discharge instructions, ensuring they are accompanied.

Other otology studies have also established effective protocols for ambulatory patients. A review of the literature reports a limited number of studies on the implementation of ERAS protocols for outpatient otolaryngology operations, aimed at reducing length of stay, cutting costs and improving postoperative pain and anxiety [6].

In summary, our study reports, for the first time in Morocco and Africa, the efficacy, safety and feasibility of pediatric outpatient cochlear implant surgery on a large cohort of patients. This approach used can even be extended to the adult population to meet the growing need for hearing rehabilitation, reduce hospital costs and reduce waiting list for surgery in busy hospital settings.

## Conclusion

Ambulatory surgery offers surgical results similar to those of conventional hospitalization, with benefits for both the patient and the healthcare system. Our findings demonstrate that overall, cochlear implant surgery can be performed safely and effectively on an outpatient basis, and its generalization to other hospitals in Morocco would allow us to better define the benefits of this type of surgery and assess its safety.

## Data Availability

No datasets were generated or analysed during the current study.
